# TiB_2_-Based Composites for Ultra-High-Temperature Devices, Fabricated by SHS, Combining Strong and Weak Exothermic Reactions

**DOI:** 10.3390/ma6051903

**Published:** 2013-05-10

**Authors:** Marta Ziemnicka-Sylwester

**Affiliations:** Division of Materials Science and Engineering, Hokkaido University, Sapporo 060-8628, Japan; E-Mail: marta.zs@eng.hokudai.ac.jp; Tel./Fax: +81-11-706-6345

**Keywords:** TiB_2_, boron carbide, ceramic matrix composites (CMCs), self-propagating high temperature synthesis (SHS), ultra-high temperature ceramics (UHTCs)

## Abstract

TiB_2_-based ceramic matrix composites (CMCs) were fabricated using elemental powders of Ti, B and C. The self-propagating high temperature synthesis (SHS) was carried out for the highly exothermic “*in situ*” reaction of TiB_2_ formation and the “tailing” synthesis of boron carbide characterized by weak exothermicity. Two series of samples were fabricated, one of them being prepared with additional milling of raw materials. The effects of TiB_2_ vol fraction as well as grain size of reactant were investigated. The results revealed that combustion was not successful for a TiB_2_:B_4_C molar ratio of 0.96, which corresponds to 40 vol% of TiB_2_ in the composite, however the SHS reaction was initiated and self-propagated for the intended TiB_2_:B_4_C molar ratio of 2.16 or above. Finally B_13_C_2_ was formed as the matrix phase in each composite. Significant importance of the grain size of the C precursor with regard to the reaction completeness, which affected the microstructure homogeneity and hardness of investigated composites, was proved in this study. The grain size of Ti powder did not influence the microstructure of TiB_2_ grains. The best properties (HV = 25.5 GPa, average grain size of 9 μm and homogenous microstructure), were obtained for material containing 80 vol% of TiB_2_, fabricated using a graphite precursor of 2 μm.

## 1. Introduction

Due to extremely high melting point, excellent corrosion resistance, low theoretical density, and excellent creep resistance boron based compounds such as TiB_2_ or B_4_C, are ideal candidates for advanced ultra-high temperature devices [1,2,3,4,5,6]. Several applications have already been considered and reported including ultra-high temperature high wear-resistance devices. However, most consideration has been devoted to their potential application as control rods [7,8] and shielding material for the nuclear industry, due to the large neutron absorption of boron atoms [4,9,10,11,12,13].

The melting point of pure TiB_2_ equals 3490 K while B_4_C melts at 2730 K. Vallauri *et al.* [14] assumed that the critical assessments of several researchers had revealed that the Ti-B-C system involves only binary compounds, since no ternary ones were found. According to Udalov [15] the two phases exhibit complete mutual insolubility of the components [16] and form simple eutectics with the lowest melting point of 2470 K corresponding to 74% B_4_C and 26% TiB_2_ [15]. A similar eutectic temperature (2495 K) was calculated by Zakaryan *et al.* [16]. Such data are approximately consistent with calculations reported by Velikanova *et al.* [17], which indicated a eutectic point at 2639 K, for 78.54 at% B. This data indicates consistently that TiB_2_-B_4_C composites are expected to sustain extremely high temperatures, beyond 2300 K, while their properties should correspond well with that of pure TiB_2_ and B_4_C.

TiB_2_ has a unique combination of properties, such as a low density of 4.52 g/cm^3^, high microhardness (34 GPa) as well as good thermal and electrical conductivity [18]. It is the hardest interstitial boride possessing a hexagonal A1B_2_-type structure [16], where boron atoms fill the trigonal prisms formed by the atoms of titanium [14]. Such an interstitial boride is a highly wear and temperature resistant structural ceramic with excellent thermal and chemical stability up to about 2000 K, therefore it remains an interesting material for ceramic composites. Due to the anisotropy of the thermal expansion coefficient, such a phase is expected to generate crack deflection in composite materials [19]. However, the anisotropy in the structure is an additional factor impeding while sintering [14].

Boron carbide, which is an interstitial carbide assigned as B_4_C, is one of the hardest materials known, ranking third behind diamond and cubic boron nitride (c-BN) [9,20,21]. Boron carbide also exhibits a set of excellent properties, especially a remarkably low density of 2.54 g/cm^3^ [8], a high melting point, and extreme hardness of 36–38 GPa [15]. Other parameters of this compound are compressive strength of 2.86 GPa, high chemical stability [7,12] and good wear and abrasion resistance [11]. It is the hardest material at temperatures above 1373 K [9,21] which melts congruently at 2723 K at a composition of 18.5 at% C. It is not surprising, because boron carbide is ordinarily a solid solution, stable over the composition range of 8.9–24.3 at% C [21], or 10.0 to 20.9 wt% C and co-exists in equilibrium when higher concentration of carbon occurs [22]. Indeed, both carbides (B_4_C and B_13_C_2_) have a rhombohedral structure (R-3m), characterized by different lattice parameters and different densities 2.51 and 2.45–2.48 g/cm^3^, respectively. The lattice parameters of B_13_C_2_ are a = b= 5.6170, c = 12.0990 Å, while for B_4_C the parameters equal: a = b = 5.620, c= 12.0990 Å. The only difference in the lattice structure is the existence of C-B-C chains in the B_13_C_2_, instead of C-C-C in B_4_C [23,24,25], while Heian *et al*. [21] concluded that there is still some uncertainty about such a complex structure which is difficult to investigate. Regardless of the unclear lattice structure, the limitation of this material is low strength (200–400 MPa) and poor fracture toughness 2–3 MPa·m^1/2^ [20]. There is currently intensive research on B_4_C in order to improve its sinterability, strength and toughness. This should guarantee appropriate use of its superior high temperature hardness [19], in a way similar to TiB_2_. Also the high temperature properties due to covalent bonds cause poor sinterability [10,20]. To overcome this issue, metal matrix composites (MMCs), have been extensively developed [4,6,18,26,27,28]. In this case, the lower melting point metal additive used as a matrix phase, especially one forming low temperature eutectics, causes significant reduction of the maximum temperature the composite could be applied, due to softening and poor creep resistance [19,26]. Indeed, ceramic matrix composites (CMCs) based on only TiB_2_ and B_4_C, are better candidates for harsh environments [20] and ultra-high temperature devices [19].

However, due to covalent bonds and small diffusion coefficients, which are advantageous for their applications [1], both TiB_2_ and B_4_C are considered as materials which create a challenge for sintering [2,7,12]. Neither B_4_C nor TiB_2_ can be obtained as a monophasic full density material, despite many methods that have been applied, such as pressure-less sintering at 2423 K [12,29], HP (Hot Pressing) [8,14,18,20,30], SPS (Spark Plasma Sintering), reaction sintering [7,14,30] *etc.* The combination of two different highly refractory materials *i.e*. TiB_2_ and B_4_C is expected to improve sinterability, since one can play the role of a sintering additive [10,31], and due to improved densification can enhance the bending strength and fracture toughness [5,6,7,32]. The addition of a second phase significantly inhibits the grain growth [14,29,30] in the microstructure and finally extends the life time under severe harsh high temperature conditions. Therefore, research efforts have turned recently to improve sinterability and fracture toughness of Ultra-High Temperature Ceramics, (UHTCs) in the Ti-B-C system.

Self-propagating high temperature synthesis (SHS), also known as combustion synthesis, is inexpensive, straightforward and an attractive method convenient for synthesis of advanced materials, such as high-refractory non-oxide ceramics [2,18,31,33] or multiphase materials [13], from elemental powders. Exploiting the self-sustaining character of a highly exothermic reaction once initiated, allows reduction of the cost of sintering, which is significant for highly refractory materials [34]. For instance, SHS can be successfully used in order to synthesize TiB_2_, due to the extremely high adiabatic temperature of over 3300 K which could be reached during the synthesis using elemental Ti + B powders under adiabatic conditions [35]. Holt *et al.* [34] who investigated the thermodynamics and kinetics of the combustion synthesis of TiB_2_ assumed that the activation energy of 539 kJ for the Ti + 2B reaction is lower than the value of 773 kJ which correspond to sintering of TiB_2_. They indicated different mechanisms for two such processes as well as limitation in sintering of TiB_2_ during combustion synthesis, despite high temperature.

Also the reaction of 4B + C = B_4_C is exothermic, however the exothermicity is very weak and the adiabatic temperature rise of 1000 K [36] is much lower than for TiB_2_ formation. Based on experimental results reported by Munir [36], the reaction cannot self-sustain unless the adiabatic temperature exceeds 1800 K. It means that the reaction has to be activated to overcome both thermodynamic and kinetic limitations of the sluggish SHS process. For B_4_C formation Xue and Munir [33] developed a model of external fields on the combustion synthesis in 1997–1998, and recently Heian *et al*. [21], as well as Zhang *et al*. [35] proposed field activated SHS for such a purpose. The last investigations assumed that when the field-activated temperature was 800 K, 900 K and 1000 K, self-propagating reaction was not possible, due to insufficient energy to activate the reaction. When the activated temperature exceeds 1100 K, the adiabatic temperature for the combustion synthesis is more than 1800 K and the reaction can self-sustain. In most cases, SHS is applied for fabrication of monophase or composite powders [32], which can be sintered using several technological processes [11,31]. SHS was employed to achieve a low energy consumption process by using strong and weak exothermicity related to the formation of TiB_2_ and B_4_C, respectively, as well as for other combinations *i.e.* TiC + SiC [37,38], or SiC-B_4_C [13]. However, by conducting the synthesis simultaneously under high pressure, the composites can be fabricated in a one stage process. It has been demonstrated that the application of pressure during or subsequent to the combustion step can significantly increase the product density [2], especially for synthesis with additives [28,39].

The purpose of the present study was to fabricate TiB_2_-B_4_C composites using elemental powders via simultaneous synthesis and densification by means of SHS-p-HIP. The SHS reaction intended to synthesize TiB_2_ was combined with pseudo-hot-isostatic pressing in order to considerably improve the densification process. The energy required for “*in situ*” B_4_C formation was ensured by a highly exothermic synthesis of TiB_2_ by means of SHS. The kinetics of SHS can be enhanced by using ultra-fine precursors, especially carbon, or additionally by mechanical activation [3,9,32]. Since the reaction velocity depends on several parameters, including composition of the green compact as well as the grain size of precursors and homogeneity of the mixture [40], these parameters are also discussed in this paper. Several authors proposed the fabrication of TiB_2_-B_4_C composites by a carbothermal reduction method [9,10], however, with respect to purity of the composites and reduction completeness, the synthesis using pure elemental powders seems to be more effective for such purposes [3]. By using elemental powders, much better purity with significant reduction of porosity was experienced [38].

## 2. Experimental Procedure

The experiments were carried out using raw elemental powders of titanium (45 μm), amorphous boron (0.8 μm), and two different carbon precursors: 10 μm and 2 μm, respectively. In order to optimize the SHS process, two series of samples with different chemical composition as well as different dispersion of powders in the initial compact were fabricated and characterized. The composition of investigated materials is shown in Table 1.

**Table 1:** Composition of compacted powders for self-propagating high temperature synthesis (SHS).

Composite	Carbon precursor (μm)	Intended TiB_2_:B_4_C ratio		Mixing/milling time (min)	Combustion
		Atomic ratio	Volume ratio		
A	10	0.96	40:60	40	No
B	10	2.16	60:40	40	Yes
C	10	5.76	80:20	40	Yes
D	2	2.16	60:40	180	Yes
E	2	3.36	70:30	180	Yes
F	2	5.76	80:20	180	Yes
G	2	12.95	90:10	180	Yes

The powders were composed in the proper weight ratio and homogenized with 2-propanol in a planetary mill. After mixing and drying, the powders were compacted and sealed in a steel can, and then the cans were fitted with a coiled heating element.

The reactions of the compacted powder mixtures were carried out “*in situ*” in the pseudo-hot isostatic device [39] via SHS under vacuum of 10 Pa. The process was initiated by resistance heating elements. It has to be emphasized, that when the reaction had started and rapid temperature increase was noted, only high pressure of 100 MPa was used, and no heating was applied after combustion. The high pressure was held for 5 min, and then reduced to 20 MPa and held for a further 10 min.

The microstructure of investigated composites was observed using optical microscopy (OM), field-emission scanning electron microscopy (FE-SEM), as well as FE-EPMA. Phase composition was determined by X-ray diffraction, (XRD) using Cu_Kα_, while hardness was investigated using Vickers hardness tester under a load of 1 kg.

## 3. Results and Discussion

### 3.1. Heat Effects during SHS Process

The combustion occurred when the intended atomic ratio of TiB_2:_B_4_C was at least 2.16. The only case when the SHS reaction was not observed was for the atomic ratio of 0.96. This effect is consistent with previous reports [33].

Each time, the initiation of SHS occurred when the temperature of the steel can covering compact reached about 900–950 K. However, the maximum temperature varied with increasing volume ratio of the components for TiB_2_ formation.

**Figure 1:**
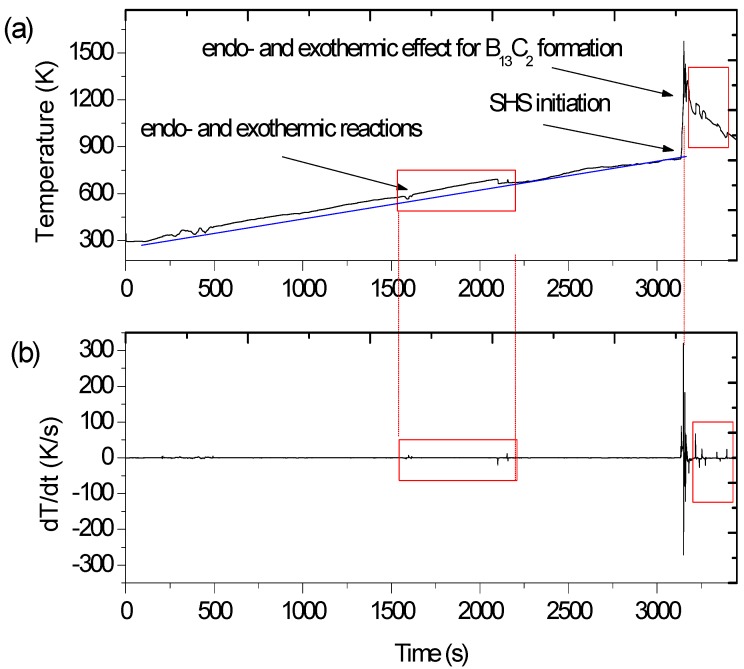
Temperature records during the SHS process held for synthesis of composite D (60 vol% of TiB_2_, C precursor 2 μm) (**a**) thermal effects related to reactions while heating before SHS initiation, and shortly after the combustion while cooling; (**b**) a derivative curve of temperature with time.

Figure 1 indicates the temperature recorded in the device during the SHS-p-HIP process which corresponds to the processing of material with the intended 60 vol% of TiB_2_ (composite D).

The temperature of the steel can (with compact inside) reached almost 1600 K in this sample, however when the concentration of TiB_2_ in the final product was higher than 80 vol% of TiB_2_ the maximum temperature caused by SHS also increased. It is believed, that the temperature inside the compact is several hundred Kelvins higher than that measured for the can sealing compacts.

The synthesis of each composite in the Ti-B-C system involved an unstable heating rate at temperatures above 720 K. Such an effect of irregular temperature increase, visible especially on the derivative curve (Figure 1b), may indicate both endothermic and exothermic reactions which occur before the initiation of SHS. The process consumed and released in turn some heat, due to phase transformations or carbon and boron solid state diffusion. A similar effect, however much more intensive, was observed after the combustion while cooling, which means that the kinetics of the boride formation was improved due to a significantly increased temperature.

### 3.2. Phase Composition of Composites after SHS

**Figure 2:**
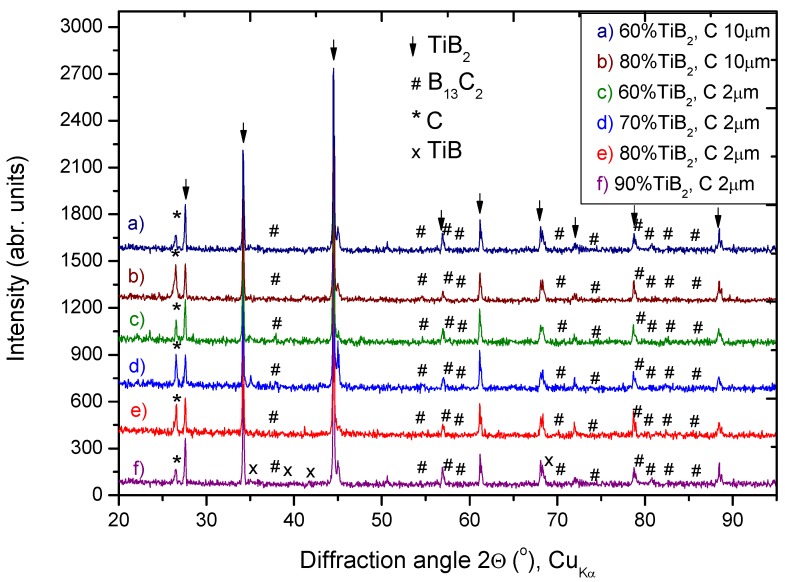
The X-ray diffraction, (XRD) patterns for two series of investigated samples (**a–b**) synthesized with C_graph_. 10 μm and milled for 40 min (composites B and C); (**c–f**) materials obtained using C precursor of 2 μm and the mixture milled for 3 h (composites D–G, respectively).

The SHS process in the Ti-C-B system brought about the formation of TiB_2_ as a predominant phase, and B_13_C_2_ as the main component of the matrix. Moreover, a small amount of unreacted C was distinguished in each sample, sometimes in negligible concentrations (Figure 2). The efficiency of boron carbide formation expressed as B_13_C_2_:C_graph_ ratio increased with a higher concentration of TiB_2_ in the composites. The heat released during the exothermic TiB_2_ synthesis as well as temperature increase with increasing concentration of such a phase. The synthesis of B_4_C requires heat to proceed, which seems to be insufficient when the TiB_2_ content is reduced. However, it should be emphasized that the accuracy of XRD is limited, especially as graphite is characterized by only one strong peak in the XRD pattern, while B_13_C_2_ and B_4_C have several small peaks in the reference pattern, as well as expected small crystallites of B_13_C_2_. Therefore quantitative analysis is characterized by low accuracy.

The results of unreacted carbon are apparently consistent with Zhang *et al*. [35], who worked on *in situ* synthesis and sintering using pulse electric current sintering. They assumed that synthesis of interstitial boron carbide cannot proceed by mean of SHS when the temperature is lower than 1100 K. That means for those experiments, boron carbide can be synthesized only for a limited period of time, until the temperature decreases below some specific temperature on cooling down. It is expected that the unreacted components, especially boron, cause deterioration in the properties of the final composites. However, if only a small amount of C is unconsumed (1–3wt%), improved sinterability is expected [41]. Similar results of B_13_C_2_ formation instead of B_4_C in the Si-B-C system were discussed by Pampuch [31], who clarified that due to spinodal decomposition of B_13_C_2_ and reaction of the secondary boron with carbon, the powder has a high chemical activity which simplifies its sintering.

Despite mechanical activation, the reaction of C with B could not be completed, because B_4_C was not detected in any composite. However, the effect of components particle size of C on the thickness of B_13_C_2_ as well as on the velocity of synthesis can be observed when considering the XRD pattern. As the average particle size of C precursor increased, the velocity of carbide formation decreased which resulted in limited consumption of C. During the long lasting process, the local extremely high temperature caused by the TiB_2_ exothermic synthesis is reduced by thermal conductivity in a non-adiabatic device and by radiation. Indeed, the temperature decreased immediately several seconds after combustion occurred. The relatively short time when the temperature is high enough for significant carbon diffusion requires a very fine carbon precursor. Otherwise, the large size graphite particles are assumed to participate as a diluent, consuming heat of the SHS reaction. Benton and Masters [8] also suggested an ultra-fine graphite precursor of 1 μm in order to synthesize and sinter B_4_C.

### 3.3. Microstructure

**Figure 3:**
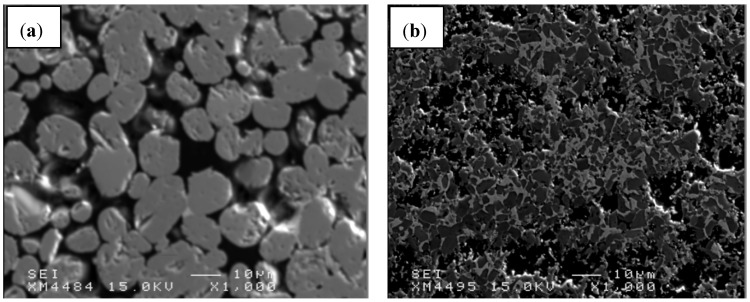
Field-emission scanning electron microscopy (FE-SEM) microstructure for composites with different TiB_2_ vol%, fabricated using graphite powder of 10 μm (**a**) 80 vol%TiB_2_, (composite C); (**b**) 60 vol%TiB_2_ (composite B).

Based on the inhomogeneous microstructure of composites synthesized using less fine C powder of 10 μm and when the mixture of elements was blended for 40 min (Figure 3), it can be assumed that significant difficulty of TiB_2_ and B_13_C_2_ formation occurred. The microstructure is characterized by essential porosity, agglomerated TiB_2_ grains and poor homogeneity, especially in the composite with 60 vol% of TiB_2_.

Several authors working on SHS investigated the effect of precursor grain size on the mechanism and velocity of heat wave propagation, including Merzhanov, Novozhilov and their coworkers [34]. In terms of TiB_2_ synthesis, the finer particle size of boron should result in a greater wave velocity [34]. However, considering the high efficiency of TiB_2_ formation, the reason for such inhomogeneous microstructures was a carbon precursor and the slow kinetics of boron carbide formation.

Therefore, in order to improve the diffusion and reaction velocity of the boron carbide synthesis, the other C precursor with average grain size of 2 μm was applied. Moreover, the powder mixture was milled using a planetary mill for 3 h, and then a second series of four samples was prepared with the intended TiB_2_ content varying from 10 to 40 vol%.

The porosity of materials fabricated by means of SHS-p-HIP was significantly reduced after using more fine carbon powder.

**Figure 4:**
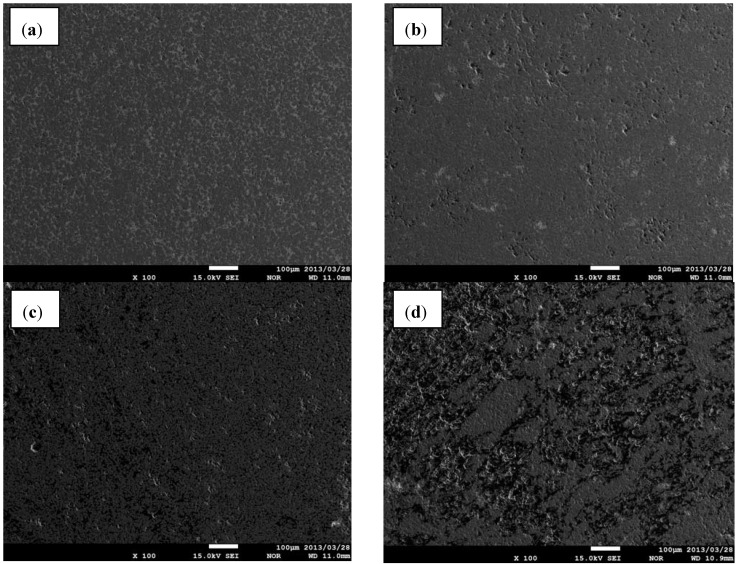
FE-EPMA microstructure (under low magnification) for composites with different TiB_2_vol% fabricated using graphite powder with the grain size of 2 μm (**a**) 90vol%TiB_2_, (composite G); (**b**) 80vol%TiB_2_ (composite F); (**c**) 70vol%TiB_2_ (composite E); (**d**) 60vol%TiB_2_ (composite D).

Considering the above microstructures (Figure 3 and Figure 4) it can be assumed, that the homogeneity of investigated composites was also significantly improved when the compact for SHS was prepared using C with the grain size of 2 μm and followed by extensive milling. Such effect is especially observed when the concentration of TiB_2_ exceeds 70vol% in the composite.

**Figure 5:**
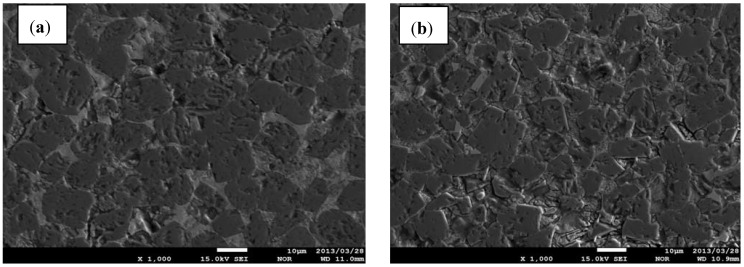

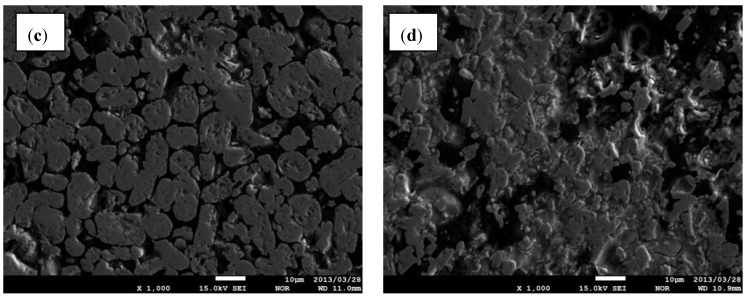
FE-EPMA microstructure (under high magnification) for composites with different TiB_2_ vol% fabricated using graphite powder with the grain size of 2 μm (**a**) 90vol% TiB_2_, (composite G); (**b**) 80vol% TiB_2_ (composite F); (**c**) 70vol% TiB_2_ (composite E); (**d**) 60vol% TiB_2_ (composite D).

The same composites were investigated using FE-EPMA under high magnification, and the microstructure is shown in Figure 5.

Significant improvement (by using precursors of finer grain size) in the homogeneity was confirmed by microstructure observations using high magnification, especially in samples with 80 and 90 vol% of TiB_2_. The FE-EPMA observation of the microstructure for composite having 90 vol% of TiB_2_ (Figure 5a) verified by EDS indicates that round grains of TiB_2_ were formed. However many defects could be observed, such as elongated pores and spallation within the grains, which were caused by too high a velocity of SHS and huge temperature gradients. Indeed, large defects were formed on cooling. Also the distribution of matrix B_4_C is not regular, because some TiB_2_ agglomerates occurred. Only 10 vol% on diluting B_13_C_2_ seems to be insufficient to avoid TiB_2_ agglomerations which makes further densification impossible.

The most homogenous microstructure with reduced porosity was observed in the material with a concentration of TiB_2_ reduced to 80 vol% (Figure 5b). The unfavorable effects of too high a velocity of SHS could be significantly decreased when the volume fraction of TiB_2_ was reduced, and a similar microstructure, although indicating more inhomogeneity, was observed at the composite with 70 vol% of TiB_2_. Finally, the microstructure becomes much worse in the composite containing only 60 vol% of TiB_2_. It may indicate a different reaction mechanism, caused by lower heat during the SHS process. The most probable reason for such a non-uniform microstructure is that the Ti grains in the compact were neither fully consumed for TiB_2_ formation nor totally melted during SHS.

**Figure 6:**
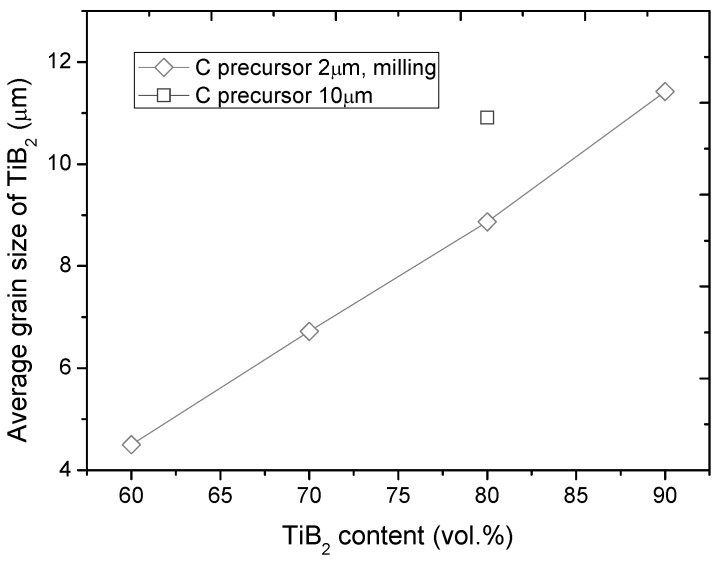
Average grain size of TiB_2_ in the microstructure of TiB_2_-B_13_C_2_ composites fabricated using C precursor of 2 or 10 μm.

Moreover, the volume fraction of TiB_2_ influenced the average grain size in the composites, which monotonically increased with increasing concentration of TiB_2_ (Figure 6).

The average grain size of TiB_2_ in the composites enlarged significantly when the vol fraction of TiB_2_ increased. Such a relationship is in good compliance with expectations, because the components for B_4_C formation (C + B) play the role of diluents from the perspective of TiB_2_ formation by means of SHS. While comparing the TiB_2_ grain size in samples fabricated using different carbon precursors it can be assumed, that diffusion of C into B which resulted in formation of B_13_C_2_, consumes heat from the exothermic TiB_2_ synthesis. The grain size of TiB_2_ was reduced when the efficiency of B_13_C_2_ formation increased. This indicates, that despite exothermicity of boron carbide formation (adiabatic temperature of 1273 K), such a reaction requires significant energy from an external source to be able to proceed.

### 3.4. Dissertation on the Reaction Mechanism

The mechanism of SHS for TiB_2_ has already been reported by several authors. However, it is a significant challenge to determine the exact mechanism for the Ti-B-C system, especially in non-adiabatic conditions under pressure. Considering both XRD data and microstructure, the most possible scenario of the reaction includes the following processes:

1. Formation of TiB, TiB_2_ nanolayers on the surface of titanium grains, by means of solid state reaction. Since the formation of TiB and TiB_2_ is strongly exothermic, locally the temperature increases immediately and the SHS process can be initiated. The study of Ti + 2B → TiB_2_ reaction has shown that this reaction begins to proceed at a noticeable rate long time before Ti starts to melt [40]. A low rate diffusion of carbon into boron is also predicted simultaneously with the preliminary formation of TiB and TiB_2_;
2. Initiation of SHS caused by heat released from the first portion of TiB and TiB_2_ causes improved diffusion which affects the accelerated formation of products. Significantly increased temperature causes melting of Ti unreacted in the first stage of the process. The boron and carbon present in the mixture can be partially dissolved in the liquid titanium and precipitate as B_13_C_2_ monolayers, along with TiB_2_ formation. The reaction rate depends on the heating rate before combustion. The slower the sample is heated and the longer it is held at high temperature before explosion, the higher the reaction rate in the run [40];
3. Migrating thin-reaction-layer mechanism followed by precipitation from a liquid phase occurs while cooling.

It is believed that this mechanism is not significantly different from the reaction of Si-B-C [31]. However the temperature onset on the profile during synthesis (Figure 1) indicated that the combustion starts before melting of Ti.

Considering the thermodynamics the process described in this paper deals with fabrication of composites *in situ*, combining strong and weak exothermic reactions. The strong exothermicity involves formation of TiB_2_ by means of SHS, where the adiabatic temperature equals approximately 3450 K. However, many researchers admitted that despite extremely high adiabatic temperatures the maximum temperature apparently observed in the proceeding SHS reaction is significantly smaller, sometimes by 1000K, than the calculated adiabatic temperature [33]. This effect is most often explained by non-adiabatic conditions, however considering the temperature range of 2300–3300 K, the heat loss by radiation should also be considered. The heat released as well as the amount of liquid titanium depend not only on initial grain size of the components in the compact but also on the concentration of Ti and other components, working as diluents. For instance, by applying a concentration of TiB_2_ reduced to 60 vol%, a weak exothermic effect was generated, as well as an essentially reduced amount of liquid titanium during the SHS process. Such conditions resulted in a nonhomogeneous microstructure and essential (4B + C) reaction incompleteness. A different reaction mechanism and significantly lower adiabatic temperature, of about 1300 K, is expected for boron carbide (B_13_C_2_). Apparently, the solid state reaction is responsible for B_13_C_2_ as well as B_4_C formation [8], so the diffusion of carbon and boron is characterized by a much smaller rate than TiB_2_ formation. It means that the reaction has to be supported by an external heat source in order to proceed. Indeed, there are two steps for formation of these composites. The first one takes place at or near the leading edge of the combustion wave, while the second occurs at or near the tailing edge of the wave. A similar technique to combine strong and weak exothermic reactions was reported by Xue and Munir [33] who carried out research with field activated combustion.

The mechanism of B_13_C_2_ formation and phase transition has become recently a subject of extensive studies [23]. More information is needed on the structure-properties relationship. It can only be assumed that decreased concentration of C in the boron carbide results in unreacted carbon as a separate phase in the composites. The consequence for high-temperature application depends on the atmosphere where the material is to be applied. The application in a protective argon atmosphere should not affect its refractoriness, but only its mechanical properties. However, applicability in a harsh oxidative atmosphere should affect both corrosion resistance and mechanical properties. In order to take maximum advantage of these materials, the predictions have to be confirmed experimentally.

### 3.5. Hardness

**Figure 7:**
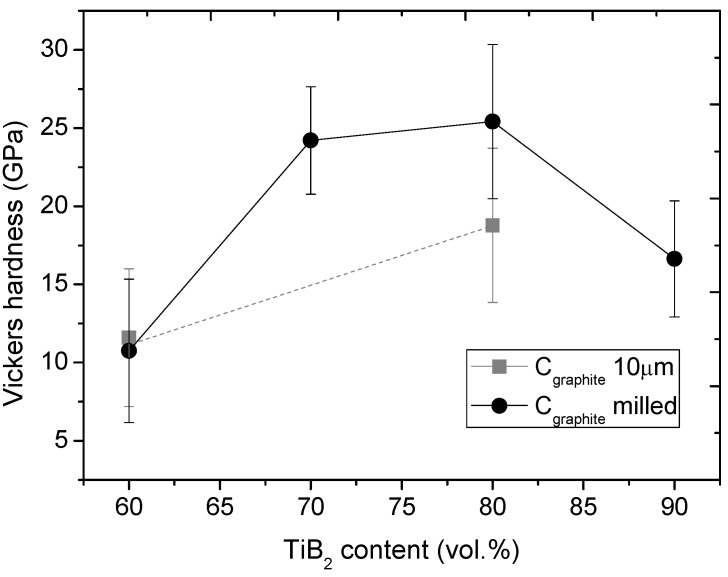
Vickers hardness of investigated composites with different TiB_2_ content, determined under a load of 1 kg.

The Vickers hardness revealed significant importance of TiB_2_ content, since the hardness varied in the wide range, from 10.7 to 25.5 GPa. Based on the Vickers hardness measurements (Figure 7), the highest hardness revealed the composite with 80 vol% of TiB_2_. The hardness of 25.5 GPa indicates, that boron carbide significantly enhanced the composite, since such a high hardness was obtained for polycrystalline material. The slightly reduced microhardness of 24 GPa, was measured when the volume fraction of B_4_C increased to 30 vol%.

When the concentration of TiB_2_ was 60 vol%, the hardness reached the lowest value of about 11 GPa with a significant spread of results, which can be seen based on the standard deviation expressed by error bars (Figure 7). The results meet good compliance with the microstructure (Figure 3b and Figure 5d), which indicated an inhomogeneous microstructure caused by low heat released from TiB_2_ products in the SHS process. The increased concentration of TiB_2_ to 70–80 vol% caused an elevated heat effect, which affected the smooth microstructure and gave better effectiveness in B_13_C_2_ formation, which means B_13_C_2_ is an appropriate sintering additive for TiB_2_. When the TiB_2_ volume fraction increased to 90%, the hardness significantly decreased to 16.6 GPa. Such an effect can be explained by TiB formation coexisting with TiB_2_, according to XRD results. Such TiB is characterized by an adiabatic temperature similar to TiB_2_, however with a much lower hardness of about 11 GPa [42], due to less covalent character of the chemical bonds. At the same time, many defects and agglomerates can be observed in the microstructure (Figure 5a,b). It has to be assumed, that too high a velocity of the SHS process for TiB_2_ is disadvantageous for the microstructure of SHS products, with an extremely large temperature gradient of 105 K/cm [13]. Indeed, the temperature gradient and mismatch in the thermal expansion coefficient generates thermally induced stress. As a result, brittleness is expected from a material with such a microstructure [1].

Considering the theoretical value of the Vickers hardness for both TiB_2_ and boron carbide, lower hardness was recorded for each investigated composite. Such differences can be caused by several factors: matrix phase consisting of B_13_C_2_, instead of B_4_C, unreacted carbon detected in each composite or defects in the microstructure, including porosity.

Based on the results reported by Niihara *et al*. [43], increased hardness should be achieved when the matrix phase consists of almost stoichiometric B_4_C. For nonstoichiometric B_4_C (B:C >4), such as B_13_C_2_, hardness decreased with increasing B content, suggesting that excess B diminishes the bond strength in the B_4_C structure. Indeed, the maximum hardness of the matrix phase could be 29 rather than 36 GPa, according to Thevenot [41]. The reduced hardness, at atomic ratio B:C <4 is attributed to free C in the microstructure. According to XRD patterns, the existence of unreacted C was confirmed in each composite.

Considering the hardness of composites synthesized using different C precursors, graphite powder of 2 μm or 10 μm, it can be assumed that finer carbon is much more effective as precursor for the synthesis of interstitial boron carbide. The hardness of the composite with 80 vol% of TiB_2_ increased from 16 to 25 GPa, by using C powder with an average grain size of 2 μm. Despite difficulty while operating with nano- and sub-micro powders, this is the only way to ensure active non-metallic reagents for SHS, and at the same time, to ensure significant reaction efficiency.

## 4. Conclusions

It has been proven that combining two reactions with different exothermicities and different mechanisms (rapid highly exothermic synthesis of TiB_2_ and slow kinetics weak exothermic formation of B_13_C_2_) can be useful to fabricate high temperature ceramic composites by means of SHS. However, synthesis of TiB_2_ is much more efficient, due to a much stronger exothermicity.

1. The exothermicity of TiB_2_ formation by means of the SHS process can be used in order to fabricate TiB_2_- B_13_C_2_ composites, when the volume fraction of TiB_2_ is above 60%;
2. Despite B and C composed in the ratio corresponding to B_4_C, other stoichiometry boride B_13_C_2_ was detected as matrix phase in each composite. Perhaps, further annealing after synthesis could fulfill the diffusion needed for full saturation with carbon. However, a limited number of experimental data on diffusion and phase transformations has been reported so far and is essential for consideration;
3. Several parameters, such as microstructure homogeneity, average grain size of TiB_2_ and hardness of the composites, proved that the grain size of C precursor is an important factor in effectiveness of B_13_C_2_ synthesis;
4. Sinterability of TiB_2_ can be essentially improved by using boron carbide as sintering additive, but also reduced TiB_2_ grain growth was observed. The average grain size of TiB_2_ decreased from 11 to 5 μm when the TiB_2_ vol% was reduced from 90 to 60 vol%;
5. The investigations revealed that the composite containing 80 vol% of TiB_2_ possessed the highest hardness of 25.5 GPa, low porosity, good homogeneity and compared to the composite with 90 vol% of TiB_2_, a more regular grain distribution, without agglomerates.
